# Empathic accuracy in individuals at ultra-high risk for psychosis: Using a task with dynamic real-life emotional stimuli

**DOI:** 10.1016/j.scog.2025.100404

**Published:** 2025-11-03

**Authors:** I.A. Meins, E.C.D. van der Stouwe, M. aan het Rot, B.E. Sportel, N. Boonstra, G.H.M. Pijnenborg

**Affiliations:** a Dept of Clinical and Developmental Neuropsychology, University of Groningen, Groningen, the Netherlands; b Dept of Psychotic Disorders, GGZ-Drenthe, Assen, the Netherlands; c University Medical Center Groningen, University of Groningen, Groningen, the Netherlands; d Dept of Clinical Psychology and Experimental Psychopathology, University of Groningen, Groningen, the Netherlands; e KieN VIP Mental Health Care Services, Leeuwarden, the Netherlands; f Department of Psychiatry, UMC Utrecht Brain Center, University Medical Center Utrecht, Utrecht, the Netherlands

**Keywords:** Ultra-high risk, Cognitive empathy, Empathic accuracy, Social functioning, Psychosis

## Abstract

**Background:**

Empathy as a key component of social cognition may be impaired in individuals at ultra-high risk (UHR) for psychosis. The Empathic Accuracy Task (EAT) was designed to address the dynamic and interactive nature of real-world social interactions. This study aimed to examine empathic accuracy (EA) in UHR individuals compared to controls and to explore the relationship between EAT and traditional empathy measures and social functioning.

**Methods:**

UHR individuals (n = 39) and healthy controls (n = 40) completed the EAT alongside the Interpersonal Reactivity Index, the Questionnaire of Cognitive and Affective Empathy, the Faux Pas Test and the Time Use Survey (TUS). Group differences in EAT performance were analyzed, and within-group correlations were examined between EAT scores and the TUS. Additionally, we investigated whether target characteristics (target gender, expressivity, and clip valence) moderated empathic accuracy.

**Results:**

No significant differences in EAT performance were found between groups. However, age emerged as a moderating factor, with older UHR individuals showing lower EA compared to younger UHR individuals and controls. Both groups performed better with positive videos and expressive targets. While UHR individuals reported lower social functioning and lower empathy scores on self-report measures, EAT performance did not correlate with these scores.

**Conclusion:**

Cognitive empathy appears preserved in UHR individuals, though subtle deficits may emerge with age. The lack of association between EA, self-reported empathy, and social functioning suggests that these approaches might assess distinct aspects of empathy, underscoring the complexity of empathy as an interpersonal process.

## 1 Introduction

Individuals at ultra-high risk (UHR) for psychosis are characterized by attenuated psychotic symptoms, brief limited intermittent psychotic episodes, or a genetic predisposition combined with functional decline (Carr et al., 2000). While a decline in social functioning is a key component of the UHR criteria (Kuis et al., 2021), and tends to worsen in individuals transitioning to psychosis (Jang et al., 2011), the underlying mechanisms of social decline remain unclear. Empathy, a core component of social cognition (Decety and Jackson, 2004), has emerged as a key area of interest in understanding social difficulties and a potential marker for UHR (Bailey et al., 2008; Henry et al., 2008; Kuis et al., 2021; Michaels et al., 2014). Research suggests that UHR individuals may already experience difficulties in empathy, which could contribute to their social difficulties (Kuis et al., 2021). However, previous studies on empathy have been criticized for failing to fully capture the complexity of empathy in social interactions (van Donkersgoed et al., 2019; Zaki and Ochsner, 2009), often relying on self-report measures or static tasks that do not reflect the dynamic and interactive nature of real-world social exchanges. This study focuses on a dynamic task with real-life emotional stimuli to measure empathy in UHR individuals (Aan Rot and Hogenelst, 2014; Lee et al., 2011; van Donkersgoed et al., 2019).

Empathy is a complex psychological process that involves integrating observation, memory, knowledge, and reasoning to gain understanding of others' thoughts and emotions (Ickes, 1997). Empathy encompasses both the ability to understand others' thoughts and feelings without necessarily experiencing those emotions oneself (cognitive empathy), and the capacity to emotionally resonate with others while recognizing those feelings as separate from one's own (affective empathy) (Decety and Jackson, 2004; Mazza et al., 2014; Michaels et al., 2014; Reniers et al., 2009; Tone and Tully, 2014; van Donkersgoed et al., 2019). Cognitive empathy involves complex processes like mentalizing and perspective taking (Mazza et al., 2014; Zurek and Scheithauer, 2017) and is closely related to theory of mind (ToM) (Chung et al., 2008; Zurek and Scheithauer, 2017), which refers to the ability to understand others' mental states. On the other hand, affective empathy includes sharing in others' emotions through embodied representations, often building from initial emotional contagion (Mazza et al., 2014; Hadjikhani et al., 2014; Zurek and Scheithauer, 2017).

While findings remain mixed and inconclusive, overall studies suggest that affective empathy is impaired to some extent in UHR individuals, whereas cognitive empathy appears largely preserved (Horan et al., 2010; Jhung et al., 2016; Kong et al., 2021; Kuis et al., 2021; Lee et al., 2011). However, the absence of cognitive empathy deficits in UHR individuals on performance-based tasks is somewhat unexpected. First, although there is evidence that impairments in cognitive empathy can occur without impairments in affective empathy (Dziobek et al., 2008; Kong et al., 2021; Mazza et al., 2014; Reniers et al., 2009; Rogers et al., 2007), the reverse, impairments in affective empathy without deficits in cognitive empathy are comparatively rare and primarily observed in psychopathy (Blair, 2005). Second, the lack of impairments on performance-based cognitive empathy measures in UHR individuals contrasts with meta-analyses showing that ToM abilities in this group are intermediate between those of controls and individuals with schizophrenia (Bora and Pantelis, 2013; Van Donkersgoed et al., 2015). Importantly, these meta-analyses showed impairments not just on rudimentary tasks for facial emotion recognition, such as the Facial Expressions of Emotion Stimuli and Tests (FEEST) and Reading the Mind in the Eyes Test (RMET), but also on slightly more advanced tasks involving context and social reasoning, such as the Hinting Task and Faux Pas Test. Given the conceptual overlap of ToM and cognitive empathy (Jankowiak-Siuda et al., 2016; Chung et al., 2008), it is possible that UHR individuals experience impairments in cognitive empathy, but these deficits are not captured by current assessment tools.

Notably, many performance-based tasks used to assess cognitive empathy have been criticized for failing to capture its real-world complexity. Tasks such as the Faux Pas Test (FPT) and RMET rely on static cues and lack the interactive, dynamic nature of social interactions, which typically involve rapidly evolving multisensory signals (Lee et al., 2011; van Donkersgoed et al., 2019). As a result, these tasks may not fully capture processes necessary for real-life cognitive empathy. Hence, broader social cognitive deficits underlying both ToM and cognitive empathy may not be detected using basic performance-based measures (Kuis et al., 2021; Montag et al., 2020). Thus, refining cognitive empathy assessment methods in UHR individuals may help detect subtle deficits, if present, that current measures might miss. Detecting cognitive empathy deficits is relevant as it may help to understand social functioning difficulties of UHR individuals and guide targeted support (Khanjani et al., 2015; Kuis et al., 2021).

A task designed to measure cognitive empathy in a more ecologically valid way than questionnaires and tasks using static cues is the Empathic Accuracy Task (EAT) (Aan Rot and Hogenelst, 2014; Lee et al., 2011; van Donkersgoed et al., 2019). The EAT involves participants continuously evaluating emotions in autobiographical stories told by ‘targets’ in video clips. Alignment between a perceiver's evaluation and the target's self-assessment generates an empathic accuracy score (EA) (Aan Rot and Hogenelst, 2014; Lee et al., 2011; van Donkersgoed et al., 2019). By using dynamic, naturalistic stimuli the EAT is thought to provide a more accurate understanding of cognitive empathy during the UHR phase than tests using static cues. Additionally, the EAT has the potential to offer insights into how empathy is influenced by target characteristics such as target gender, expressiveness, and clip valence (van Donkersgoed et al., 2019). Thus, the EAT could enhance our understanding of empathy and social decline in the UHR phase, aid in developing more effective and individualized therapeutic approaches and potentially improve the prediction of transition to psychosis.

Research utilizing the EAT in patients with schizophrenia showed lower EA in patients compared to controls (Lee et al., 2011; van Donkersgoed et al., 2019). Additionally, the EAT was used to investigate the role of several target characteristics including, expressivity, (i.e. how expressive the targets were), target gender and emotional valence. These studies found greater differences in accuracy between groups for highly expressive targets, with patients benefiting less from high expressiveness (Lee et al., 2011; van Donkersgoed et al., 2019). Given that UHR individuals score lower on emotion recognition than controls (Seo et al., 2020), they may similarly struggle to utilize higher expressiveness in empathic tasks (8,10).

Our hypotheses were as follows: 1) UHR individuals will score lower on the EAT compared to controls; 2) UHR individuals will benefit less from target expressivity than controls. In addition to these primary hypotheses, we explored how the target characteristics of target gender and clip valence influence empathic accuracy in UHR individuals.

Finally, to complement performance-based EA scores, we examined coherence with self-reported empathy — as measured by the *Interpersonal Reactivity Index (IRI)* and the *Questionnaire of Cognitive and Affective Empathy (QCAE)* — and explored the relationship between EA and social functioning. Insights from these analyses may clarify mechanisms underlying (social) difficulties UHR individuals experience, potentially aiding early identification and intervention efforts.

## 2 Methods

### 2.1 Participants

Baseline data were extracted from the MERIT study, a randomised controlled trial to investigate the effectiveness of a metacognitive therapy (de Jong et al., 2019). The UHR group consisted of 39 individuals with a UHR status, aged between 15 and 35 years old, all receiving mental health care. The healthy control sample contained 40 people, aged between 19 and 74 years old (for demographics, see Table 1.)

**Table 1:** Demographic variables.

Variable	Controls n = 40	UHR group *n* = 39
Gender female, n (%)	7 (17)	22 (56)
Age in years, mean, (SD), range	39.6 (13.1), 19–74	21.9 (5.7), 15–35
Level of education, mean (SD)	5.23 (0.9)	4.6 (1.3)

UHR participants were recruited from two mental health care facilities in the Netherlands: GGZ Drenthe and GGZ Friesland. All eligible patients were invited to fill out the Prodromal Questionnaire 16 at the start of their treatment as a part of routine care (Ising et al., 2012). Patients scoring a 6 or higher were invited for further assessment. The Comprehensive Assessment of At Risk Mental State (CAARMS) (Yung et al., 2005) was used to determine if the UHR criteria were met. Participants had to be able to give informed consent as determined by their therapist or case manager. Exclusion criteria included co-morbid neurological pathology, severe drug abuse/substance dependence, or an IQ score < 70. The healthy control group consisted of individuals who had never received a psychiatric diagnosis. They were recruited through social media channels and flyers in the area. The control group was older than the UHR group as it was recruited alongside the MERIT trial, which involved patients with chronic schizophrenia who are typically older (Kuis et al., 2021).

### 2.2 Instruments

#### 2.2.1 Empathy measures

Empathic Accuracy Task: The Dutch adaptation of the EAT (Aan Rot and Hogenelst, 2014; Zaki et al., 2008) was used to measure cognitive empathy. To accommodate the overall assessment within a 2-hour limit, a condensed version of the task was developed, selecting four from the original 40 videos (van Donkersgoed et al., 2019). These videos featured four distinct target individuals—two male and two female—each sharing either a positive or negative personal story. The selection of clips for each participant ensured a balance of target gender and emotional valence. Participants used a dial to rate the emotional tone (from positive to negative) in videos where an individual shared a personal anecdote. Pearson correlation coefficients were used to measure the congruence between participant scores and the emotional ratings provided by the targets. The expressiveness level of the targets was determined through their scores on the Berkeley Expressivity Questionnaire (Gross and John, 1997).

Interpersonal Reactivity Index. The IRI is a self-administered survey with 28 items, organized into four subscales of empathy (Davis, 1980). These subscales are ‘perspective taking’ (alpha = 0.72), ‘fantasy’ (alpha = 0.75), ‘empathic concern’ (alpha = 0.68), and ‘personal distress’ (alpha = 0.78) as reported in Davis, 1980. Each of the four subscales includes seven statements. Respondents are asked to rate how well each statement reflects their own feelings on a five-point Likert scale, ranging from 0 (does not describe me well) to 4 (describes me very well). Higher scores indicate greater levels of empathy. In the analysis, each of the four scales of the IRI was examined independently, as the IRI was designed for four distinct empathy facets and does not align well with a two-factor model of cognitive and affective empathy (Chrysikou and Thompson, 2015). However, personal distress and empathic concern are generally considered components of affective empathy while fantasy and perspective taking are considered components of cognitive empathy (Chrysikou and Thompson, 2015).

Questionnaire of Cognitive and Affective Empathy. The QCAE is a self-report questionnaire to assess cognitive and affective empathy through 31 items rated on a four-point Likert scale (Reniers et al., 2011). Higher scores signify increased reported empathy. The QCAE is divided into five sections: Perspective Taking and Online Simulation for Cognitive Empathy and Emotion Contagion, Proximal Responsivity and Peripheral Responsivity for Affective Empathy. In this study, composite scores were calculated separately for cognitive (α = 0.82) and affective empathy (α = 0.79) by combining relevant subscales from the QCAE (Kuis et al., 2021; Reniers et al., 2011).

Faux Pas Task. The Faux Task is a performance-based assessment of cognitive empathy (Baron-Cohen et al., 1999; Stone et al., 1998). The test comprises five stories featuring a faux pas and five without. The experimenter reads the narratives aloud, after which participants are questioned about whether a character in the story made an awkward comment (Faux Pas cognitive) and if this comment resulted in feelings of sadness and embarrassment among others in the narrative (Faux Pas affective). Both components are designed to test cognitive empathy (Kuis et al., 2021) but were used separately in the analysis.

#### 2.2.2 Social functioning

Time Use Survey (TUS). The TUS (short form) is a semi-structured interview that assesses the participant's activities over the preceding month (Lader et al., 2006). The TUS has been found acceptable in individuals with and at risk of psychosis (Hodgekins et al., 2015). Participants monitor the frequency and duration of their engagement in activities (e.g., work, education, volunteering, social activities, sleep). For each activity, a weekly average in minutes was calculated. Additionally, a cumulative weekly hour's metric is calculated combining time spent on economically productive and voluntary tasks, education, household duties, childcare, and structured activities. Its application in prior studies involving individuals with schizophrenia has shown it to be both practical and well-received (Hodgekins et al., 2015). In line with Kuis et al., 2021, this study used structured and economic activities separately as an indicator of social functioning.

### 2.3 Procedure

Upon fulfilling the UHR criteria, patients were briefed about the study and invited to participate. Following a detailed explanation of the research, written consent was obtained from all participants and the parents or guardians of those under 18 years of age. The assessment consisted of a two-hour session that included the EAT, three interviews, six questionnaires, and three neurocognitive tasks.

The study received approval from the regional medical ethics committee (METc2014.279). The ethics committee of the Psychology Department at the University of Groningen approved the comparison group study (ppo-013-109). Qualified assessors, holding at least a Bachelor of Science degree in psychology, administered all assessments.

### 2.4 Analysis

Independent *t*-tests were performed to compare mean age and education across groups, while a Chi-square test was used to examine differences in participant gender. EAT data were analyzed using multilevel models with maximum likelihood estimation. Demographic variables that differed between groups were included in the model as covariates to account for potential confounding factors. Level 1 EAT scores, i.e., correlations between target and perceiver calculated per perceiver per video clip, were transformed into Fishers z scores for the analysis. Our primary analysis investigated the overarching differences between the UHR group and the control group, identifying the group as a level 2 predictor. Additional models examined whether the valence of the videos, target gender, or target expressivity (all at level 1) moderated the effects of the group. Each model used a mixed-effects model with random intercepts for both perceivers and targets. Degrees of freedom were estimated using Kenward-Roger method (Kenward and Roger, 1997). Multilevel models were run in R. Cohen's d values were calculated from the fixed effect estimates.

Average person-level EAT scores were also calculated. These scores were utilized to calculate Pearson correlation coefficients with other empathy measures and the TUS in UHR individuals, to investigate the relationship between EA and traditional empathy assessments and social functioning. To account for multiple correlation tests, Bonferroni-corrected significance thresholds were applied.

## 3 Results

### 3.1 Demographics and general cognition

Significant differences were found in participant age and participant gender between the UHR group and the control group. The UHR group consisted of a higher proportion of female participants: χ^2^(1, *N* = 79) = 12.87, *p* < 0.001, and lower age: t (77) = 7.77, p < 0.001 compared to the control group. No significant differences in level of education were found between groups. Out of 43 UHR patients initially recruited, 39 completed the EAT. In the healthy control group, 41 participants were recruited, with one participant missing EAT data. All analysis pertains to these individuals.

Demographic variables are depicted in Table 1.

### 3.2 Empathic Accuracy Task

#### 3.2.1 Group differences in empathic accuracy and the role of gender and age

In the UHR group, the mean Fisher *Z*-transformed EA score was 1.08 (SD = 0.72, min = −2.04, max = 2.03), while in the control group, it was 1.16 (SD = 0.51, min = −0.23, max = 2.22). The average EA (i.e., correlation between participant scores and the ratings provided by the targets themselves) was *r* = 0.51 for the UHR group and *r* = 0.52 for the healthy control group.

To examine factors influencing EA, several multilevel models were conducted (see Analysis section for details). An extensive summary of all models and results is presented in Table 2.

**Table 2:** Summary results models.

Model	Predictors	F	p
Models: Demographics	Group	F(1, 75) = 0.76	0.38
	Age	F(1, 75) = 0.26	0.61
	Gender	F(1, 75) = 0.93	0.34
	Age	F(1,73) = 0.10	0.76
	Gender	F(1,73) = 0.75	0.39
	Group	F(1,70) = 0.20	0.66
	Age	F(1,71) <0.01	0.97
	Gender	F(1,72) = 0.45	0.50
	Group	F(1,70) = 4.02	0.05
	Age	F(1,71) = 3.46	0.07
	Group × age	F(1, 71) = 6.67	0.01*
	Group	F(1,70) = 2.56	0.11
	Gender	F(1,71) = 1.22	0.27
	Group × Gender	F(1,70) = 3.31	0.07
Models: Clip Valence	Valence	F(1, 206) = 25.75	<0.001***
	Valence	F(1, 206) = 25.87	<0.001***
	Group	F(1,75) = 0.89	0.35
	Valence	F(1,205) = 25.81	<0.001***
	Group	F(1,267) = 0.01	0.91
	Valence × group	F(1,221) = 0.21	0.64
	Valence	F(1,206) = 25.78	<0.001***
	Age	F(1,75) = 0.29	0.59
	Valence	F(1,206) = 25.95	<0.001***
	Gender	F(1,76) = 1.13	0.29
	Valence	F(1,206) = 25.87	<0.001***
	Group	F(1,72) = 0.59	0.45
	Age	F(1, 72) <0.01	0.93
	Valence	F(1,206) = 25.98	<0.001***
	Group	F(1,72) = 0.33	0.57
	Gender	F(1,73) = 0.57	0.45
	Valence	F(1, 206) = 25.98	<0.001***
	Group	F(1, 70) = 0.23	0.63
	Age	F(1, 71) = 0.00	0.95
	Gender	F(1, 72) = 0.56	0.46
	Target gender	F(1, 226) = 0.89	0.35
Models: Target Gender	Target Gender	F(1, 226) = 0.88	0.35
	Group	F(1,76) = 0.36	0.56
	Target Gender	F(1,225) = 0.88	0.35
	Group	F(1,267) = 0.12	0.91
	Target Gender × group	F(1, 225) <0.01	0.94
	Target Gender	F(1, 226) = 0.89	0.35
	Age	F(1, 75) = 0.10	0.76
	Target Gender	F(1, 226) = 0.89	0.35
	Gender	F(1, 76) = 0.53	0.47
	Target Gender	F(1,226) = 0.88	0.35
	Group	F(1,72) = 0.28	0.60
	Age	F(1,72) = 0.01	0.91
	Target gender	F(1,226) = 0.89	0.35
	Group	F(1,72) = 0.11	0.73
	Gender	F(1,72) = 0.30	0.59
	Target gender	F(1, 226) = 0.88	0.35
	Group	F(1, 70) = 0.10	0.75
	Age	F(1, 71) = 0.01	0.93
	Gender	F(1, 71) = 0.29	0.59
	Target Expressivity	F(1, 235) = 130.57	<0.001***
Models: Target Expressivity	Target Expressivity	F(1, 235) = 130.78	<0.001***
	Group	F(1,76) = 0.76	0.39
	Target Expressivity	F(1,234) = 130.76	<0.001***
	Group	F(1,81) = 0.46	0.50
	Target Expressivity × Group	F(1,234) = 0.70	0.37
	Target Expressivity	F(1, 235) = 130.67	<0.001***
	Age	F(1,76) = 0.27	0.61
	Target Expressivity	F(1, 235) = 130.44	<0.001***
	Gender	F(1,75) = 0.59	0.45
	Target Expressivity	F(1, 234) = 130.76	<0.001***
	Group	F(1, 72) = 0.48	0.49
	Age	F(1, 72) <0.01	0.95
	Target Expressivity	F(1, 234) = 130.61	<0.001***
	Group	F(1, 73) = 0.39	0.53
	Gender	F(1, 73) = 0.22	0.64
	Target Expressivity	F(1, 234) = 130.59	<0.001***
	Group	F(1, 70) = 0.25	0.62
	Age	F(1, 71) < 0.01	0.97
	Gender	F(1, 72) = 0.22	0.64
	Time Use: Economic	F(1, 75) = 0.86	0.77
Models: Time Use Economic	Group	F(1, 74) = 0.67	0.42
	Time Use: Economic	F(1,74) < 0.01	0.98
	Group	F(1,73) = 0.59	0.44
	Time Use: Economic	F(1,74) = 0.02	0.88
	Group × Time Use: Economic	F(1,74) = 0.54	0.47
	Time Use: Structured	F(1, 73) = 0.21	0.65
Models: Time Use Structured	Group	F(1, 74) = 0.79	0.38
	Time Use: Structured	F(1,72) = 0.25	0.62
	Group	F(1,73) = 0.77	0.38
	Time Use: Structured	F(1,74) = 0.13	0.71
	Group × Time Use: Structured	F(1,74) < 0.01	0.95

Note, all models include random intercepts for perceiver and target to account for variability at these levels.

Neither age nor gender significantly predicted EA scores, indicating that these demographic factors do not independently explain variability in EA.

**Fig. 1:**
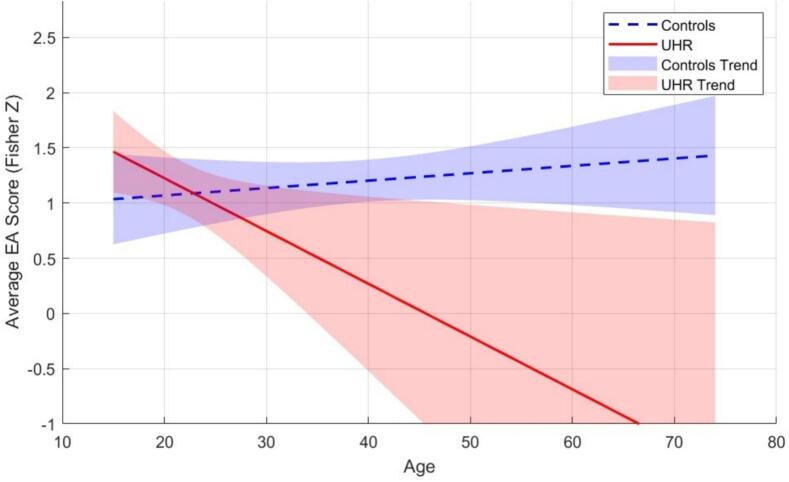
Empathic accuracy (Fisher Z) across age in UHR and controls. Note, the control group's broader age range leads to extrapolated regression estimates beyond the observed UHR range.

When group was the only predictor, no significant effect was found, suggesting that UHR individuals do not differ from controls in EA. Additionally, interactions between group and age or gender were tested. No significant group × gender interaction was found. The interaction between group and age showed significance, suggesting that the effect of age varied between groups. Follow-up analyses showed that the slope of age for the UHR group was significantly negative (estimate = −0.04, *t* = −2.60, *p* = 0.01), while the slope for the control group was not significantly different from zero (estimate <0.01, *t* = 0.95, *p* = 0.34). This indicates that EA scores were higher among younger individuals in the UHR group, whereas in the control group, EA scores were unrelated to participant age (see Fig. 1).

#### 3.2.2 The role of clip valence in empathic accuracy

A significant effect on EA scores was found when Valence was the only predictor, indicating that participants were more accurate in rating positive video clips compared to negative clips. Adding group to the model did not affect the valence effect. The group × valence interaction was not significant, indicating that both groups showed similar improvement in EA with positive clips. Adding either participant age or participant gender as covariates to the models did not meaningfully change these results. These findings show a robust effect of clip Valence.

#### 3.2.3 The role of target gender in empathic accuracy

When target gender was the only predictor, it did not significantly explain variability in EA. In subsequent analyses that were comparable to the analyses presented under 3.2.2 but with clip valence replaced by target gender, there were no significant results. This indicates that target gender does not explain variability in EA and that nonsignificant results for group could not be explained by a moderating role of target gender.

#### 3.2.4 The role of target expressivity

A strong main effect of target expressivity was found, showing that more expressive targets were easier to interpret. Adding group did not change the effect, and the group × expressivity interaction was not significant, suggesting that both UHR individuals and controls benefit equally from high expressivity. Adding gender or age did not change the effect.

### 3.3 Correlations among empathy measures

We examined correlations between aggregated EAT scores and subscales of traditional empathy assessments in both controls and UHR individuals. In the UHR group, a moderately negative correlation was found with the Perspective taking subscale of the IRI (*r* = −0.33, *p* = 0.04). However, after applying a Bonferroni correction for multiple comparisons (α = 0.0031), no correlations remained statistically significant. Results are depicted in Table 3.

**Table 3:** Correlations between EAT and self-report/performance-based empathy measures in UHR and control groups.

Questionnaire	Controls Correlation with EAT data (r,p)		UHR group Correlation with EAT data (r,p)	
Faux-pas cognitive	0.20	0.26	−0.06	0.71
Faux-pas cognitive/affective	0.31	0.07	−0.20	0.23
QCAE cognitive	0.07	0.68	−0.24	0.13
QCAE affective	−0.22	0.17	−0.13	0.43
IRI PT	0.09	0.59	−0.33	0.04
IRI FS	−0.25	0.12	−0.17	0.30
IRI EC	0.06	0.72	−0.31	0.06
IRI PD	−0.18	0.27	−0.18	0.28

### 3.4 Empathy accuracy task and social functioning

The relationship between EA and social functioning, measured using the Time Use Survey's Economic and Structured subscales, was investigated. Correlation analyses revealed no significant associations in either the control or UHR group. Results are depicted in Table 4.

**Table 4:** Pearson correlations between EA and constructive and economic subscales of the time use survey.

Questionnaire	Controls Correlation with EAT data (r,p)		UHR group Correlation with EAT data (r,p)	
TU economic	0.19	0.25	−0.07	0.67
TU structured	0.08	0.64	0.06	0.69

Additionally, exploratory analyses incorporating group, social functioning subscale, and their interactions as predictors of EA showed no significant main effects or interactions for either subscale (see Table 2). These findings suggest nonsignificant results for group could not be explained by a moderating role of social functioning.

## 4 Discussion

This study examined empathic accuracy using the EAT in UHR individuals compared to general population controls. We hypothesized that UHR individuals would exhibit lower EA scores and benefit less from target expressivity than controls. Contrary to our expectations, group differences in EA were not statistically significant, also when taking target expressivity into account, as well as other potential moderators (participant age and gender, clip valence, target gender, social functioning). These findings contrast with previous reports of EA impairments in schizophrenia (Harvey et al., 2013; Lee et al., 2011; van Donkersgoed et al., 2019), suggesting that empathic difficulties may not yet be present or detectable in the UHR phase.

Our findings align with prior research showing that UHR individuals show no empathy difficulties in structured, performance-based tasks amidst impairments on self-report empathy (Konstantakopoulos et al., 2014; Kuis et al., 2021; Murphy and Lilienfeld, 2019). Additionally, the EAT was not associated with other performance-based measures of cognitive empathy (e.g., Faux Pas). This discrepancy suggests that these approaches might assess distinct aspects of empathy and underscores that subjective experiences of empathy may not align with the observable abilities measured by the EAT.

The absence of convincing impairments in EA appears inconsistent with robust findings of ToM deficits in UHR individuals (Bora and Pantelis, 2013; Van Donkersgoed et al., 2015). A possible explanation is that UHR individuals may compensate for subtle deficits in EA through cognitive strategies, such as increased self-monitoring (Hou et al., 2022), combined with reliance on the emotional and contextual cues provided by the EAT— such as vocal tone, non-verbal behaviours, and narrative content. By utilizing richer, more ecologically valid stimuli, the EAT may support these compensatory mechanisms, enabling UHR individuals to perform on par with controls, despite scoring lower on rudimentary social cognitive tasks (Bora and Pantelis, 2013; Hou et al., 2022; Seo et al., 2020; Van Donkersgoed et al., 2015). Neuroimaging studies further support this hypothesis, showing increased neural activity during social cognitive tasks in UHR individuals, even without observable behavioural differences (Bartholomeusz et al., 2018; Hou et al., 2022). These findings suggest that UHR individuals may rely on additional cognitive resources or contextual cues to match performance with healthy controls.

The efficacy of these compensatory strategies in UHR individuals may depend on both the cognitive demands of the task and the extent of impairments. Research has suggested that deficits in ToM may become pronounced as task complexity increases (Van Donkersgoed et al., 2015). This raises the possibility that compensation may only be possible up to a certain threshold. Compared to traditional ToM tasks, the EAT may impose lower explicit cognitive demands by relying on continuous, real-time emotion tracking rather than explicit reasoning about others' mental states. This distinction could explain why UHR individuals perform similarly to controls on the EAT but show deficits on more structured, perspective-taking-based ToM tasks. Supporting this, intelligence has been shown to correlate with traditional performance-based tasks like the Faux Pas Test (Van Donkersgoed et al., 2015), but not with the EAT (Ponnet et al., 2005). This challenges earlier assertions that intelligence could mitigate difficulties in conceptual perspective-taking strategies (Ponnet et al., 2005; Prior et al., 1990; Yirmiya and Shulman, 1996), but also suggests that the EAT captures a distinct, perhaps more intuitive, facet of empathic processing that may be less reliant on cognitive resources.

Moreover, exploratory analyses suggested that age might moderate EA in UHR individuals, with older UHR participants showing a mild impairment in EA, while younger UHR individuals perform comparably to healthy controls. This significant age-related pattern suggests subtle impairments might emerge in UHR participants at the upper end of the age range, potentially reflecting symptom progression that can no longer be fully compensated for. Previous research has shown that empathic impairments are more pronounced in chronic schizophrenia patients compared to first-episode patients (Achim et al., 2011). Considering this progression, EA impairments during the UHR phase may emerge only in later stages. However, without longitudinal and clinical data, it remains unclear whether this pattern reflects symptom progression or other moderating factors. Additionally, the control group's wider age range (19–74) means the regression line extrapolates beyond the UHR range (15–35), warranting caution in interpretation.

While higher target expressivity was associated with higher EA for both groups, this did not differ between controls and UHR individuals. This suggests that low expressivity did not disproportionately affect UHR individuals, and might question the extent that the richness of the EAT explains compensatory performance in UHR individuals. However, expressivity is assessed through self-report and may not reflect all expressive modalities such as tone of voice or speech content as provided by the EAT (Wever et al., 2022). Additionally, in this study we used 4 targets which reduced the diversity of expressivity scores compared to the original EAT (Lee et al., 2011; Wever et al., 2022). Considering these limitations, our findings suggest that, although some (older) UHR individuals might exhibit subtle deficits in EA, at least in early stages they perform on par with controls, possibly due to compensatory mechanisms.

Furthermore, the lack of association between EA and social functioning in our study contributes to the broader debate on the link between social cognitive capabilities and psychosocial outcomes. Our findings align with previous research suggesting that social cognitive abilities are not always straightforwardly linked to psychosocial functioning (James et al., 2017; Lysaker et al., 2021). As functioning within UHR is generally heterogeneous (Jang et al., 2011), we tested social functioning as a potential moderator but found no evidence for such an effect. However, social functioning assessments in UHR and schizophrenia are often crude and lack sensitivity and small changes in social functioning may go undetected (Burns and Patrick, 2007). Additionally, it has been hypothesized that social cognitive capacity needs to be below a threshold to significantly affect social functioning (Pijnenborg et al., 2009). Whether the lack of interaction between EA and social activities is a product of the TUS, the social functioning levels in this sample still being relatively preserved despite being lower than controls, or whether EA plays only a limited role in social functioning requires further investigation.

### 4.1 Limitations

An important limitation of this study is that we used a shortened version of the EAT, with only four videos (van Donkersgoed et al., 2019), which may not fully capture the complexity of empathic accuracy as assessed by the original EAT. However, previous research using this adaptation suggests it can still detect group differences in empathic accuracy (van Donkersgoed et al., 2019). Nonetheless, caution is warranted when comparing our findings with studies using different EAT versions. Also, our sample had large differences in age and gender between the control and UHR groups. Although these factors were statistically controlled for, residual influences cannot be fully ruled out. Furthermore, the low specificity of the UHR criteria likely resulted in a sample with varying impairments, including cognitive deficits, autism, or personality disorders (Kuis et al., 2021; van Os and Guloksuz, 2017). For example, it has been shown that the number of autism traits is negatively associated with EAT performance (Aan Rot and Hogenelst, 2014). This variability complicates the detection of specific effects and may contribute to inconsistencies across studies, making comparisons between findings challenging (Kuis et al., 2021).

## 5 Conclusion

This study explored cognitive empathy in individuals at ultra-high risk for psychosis using an abbreviated Dutch adaptation of the EAT. Although we found no overall differences in empathic accuracy between UHR individuals and controls, subtle impairments were observed in older UHR individuals. Based on our findings and existing literature, we propose that subtle deficits in empathic capabilities may be present but can be sufficiently mitigated in some younger UHR individuals. The absence of associations between empathic accuracy, several other empathy tools, and social functioning impairments underscores the complexity of empathy as an interpersonal process. Clear operational definitions and a coherent model of empathy are needed.

## CRediT authorship contribution statement

**I.A. Meins:** Visualization, Writing – review & editing, Formal analysis, Writing – original draft. **E.C.D. van der Stouwe:** Writing – review & editing. **M. aan het Rot:** Methodology, Data curation, Writing – review & editing. **B.E. Sportel:** Writing – original draft, Writing – review & editing, Conceptualization. **N. Boonstra:** Writing – review & editing. **G.H.M. Pijnenborg:** Funding acquisition, Writing – review & editing, Conceptualization, Supervision.

## Declaration of Generative AI and AI-assisted technologies in the writing process

During the preparation of this work, the author(s) used ChatGPT to assist in the writing process. After using this tool, the author(s) reviewed and edited the content as needed and take full responsibility for the content of the published article.

## Funding information

This work was partly supported by a research grant from 10.13039/501100003142Fonds NutsOhra. No funding body agreements apply.

## Declaration of competing interest

The authors declare that they have no conflicts of interests.
